# Telehealth Use by Home Health Agencies Before, During, and After COVID‐19

**DOI:** 10.1111/1475-6773.14645

**Published:** 2025-05-22

**Authors:** Dana B. Mukamel, Debra Saliba, Heather Ladd, Melissa A. Clark, Michelle L. Rogers, Cheryl Meyer Nelson, Marisa L. Roczen, Dara H. Sorkin, Jacqueline S. Zinn, Peter Huckfeldt

**Affiliations:** ^1^ iTEQC Research Program, Department of Medicine, Division of General Internal Medicine University of California—Irvine Irvine California USA; ^2^ UCLA Borun Center at David Geffen School of Medicine Los Angeles California USA; ^3^ Veterans Administration GRECC Los Angeles California USA; ^4^ RAND Corporation—Health Care Santa Monica California USA; ^5^ Brown University School of Public Health Providence Rhode Island USA; ^6^ Survey Research Center Brown University School of Public Health Providence Rhode Island USA; ^7^ Healthcare Technology and Direct Healthcare Service Industries Scituate Massachusetts USA; ^8^ Abt Associates Rockville Maryland USA; ^9^ Fox School of Business Temple University Philadelphia Pennsylvania USA; ^10^ Division of Health Policy and Management University of Minnesota School of Public Health Minneapolis Minnesota USA

**Keywords:** COVID‐19, home health, long‐term care, payment, technology diffusion, telehealth

## Abstract

**Objective:**

To examine telehealth adoption and discontinuation by home health agencies (HHAs) during the COVID‐19 pandemic in the context of telehealth pre‐pandemic diffusion into the industry and its continued use once the pandemic abated.

**Study Setting and Design:**

HHAs nationally, serving the most patients with dementia (averaging 33% of the agency's patients) were surveyed during October 2023 to November 2024. Key variables included the agency's adoption and discontinuation of specific telehealth technologies by year, the reasons for discontinuation, and the reasons for not adopting any telehealth technology, either before or during the pandemic.

**Data Sources and Analytic Sample:**

Data were collected via a web‐based survey with telephone follow‐ups. We received 791 responses (37% response‐rate) and provide descriptive statistics of responses and graphics.

**Principal Findings:**

By 2019, prior to COVID‐19, 183 (23%) of HHAs used telehealth, increasing to 446 (56%) by 2021. Growth occurred mainly in virtual visits. Of those HHAs adopting telehealth, 96 (19%) discontinued use later in the pandemic. Key concerns were about the appropriateness of the patient population and reimbursement.

**Conclusions:**

Patterns of adoption and discontinuation suggest that COVID‐19 interrupted the innovation diffusion process of telehealth into home health. Telehealth's future will depend on information about cost‐effectiveness and Medicare reimbursement policies.

**Summary**

- What is known on this topic?
  ○ All studies to‐date are focused on the clinical effectiveness or cost‐effectiveness of telehealth in specific uses in home health.
  ○ All studies of home health use of telehealth to date are small scale, limited populations, and limited to specific diagnoses and technologies.
- What this study adds?
  ○ This is the first national, large scale study of telehealth use by home health agencies examining both adoption and discontinuation of the technology, and the reasons for these actions.
  ○ This is the only study to address this issue prior to, during, and post COVID‐19.
  ○ Only 23% of agencies used telehealth before COVID‐19; adoption peaked at 65%, but 14% of HHAs discontinued telehealth by 2024 due to lack of reimbursement or inappropriateness for patients.

## 1 Introduction

Ordinarily, home health agencies (HHAs) provide services in patients' homes, including skilled nursing care, physical, occupational, and speech therapies, and medical social work—all ordered by a physician's care plan and reflecting the regulations and reimbursement policies of the Centers for Medicare & Medicaid Services (CMS) [1]. COVID‐19 presented a challenge to this model, as patients were reluctant to allow care providers (e.g., nurses, therapists) into their homes and care providers sought to minimize in‐person contacts to reduce disease transmission, preserve scarce personal protective equipment, and manage staffing shortages. To accommodate this public health emergency, CMS allowed HHAs more flexibility to use telehealth, replacing some home visits if approved by the physician; although, unlike its policies regarding other providers such as hospitals and individual practitioners, it did not change the home health episode‐based payment system to reimburse HHAs for services provided via telehealth [2, 3].

Prior studies of telehealth use by HHAs are limited in scope, primarily focused on questions of its clinical and cost effectiveness. Most are small clinical studies examining the use of telehealth for specific diagnoses and specific telehealth technologies and services, studying a small number of agencies and small populations [4, 5, 6, 7, 8, 9, 10]. The extent to which telehealth has diffused into HHAs prior to the COVID‐19 pandemic, and whether this diffusion has accelerated during the pandemic, remains unknown. To answer these questions, we surveyed a national sample of HHAs on their adoption of telehealth, the timing of the adoption, discontinuation of use if it happened and its timing, and the reasons for both decisions to discontinue or not to adopt telehealth. In this Brief, we present the data collected from the survey and discuss its policy implications.

## 2 Methods

### 2.1 Data

We fielded a web‐based survey to 2135 HHAs about telehealth adoption and discontinuation between October 2023 and November 2024. The population was defined as the top 20th percentile of HHAs in the country in terms of their percentage of patients with dementia (on average 33% of patients) and included HHAs in all states except North Dakota. We received responses from all states except Iowa. A survey invitation with a QR code for an online survey and an offer of a $50 incentive was mailed to the head of the HHAs, accompanied by letters of support from both the National Association for Home Care & Hospice and the Forum of State Associations of Home Health Agencies. These were followed by up to 10–13 phone calls to nonresponders (see Supporting Information 1 for more details.). As the questions were about the organization and not persons, the study was considered a nonhuman subject study by the PI's Institutional Review Board.

The survey included questions about: (1) whether or not the agency adopted telehealth services, (2) the types of services adopted (see Supporting Information 2 for the list of services), (3) years of adoption and discontinuation (in detail by specific technology between 2018 and 2021), and (4) reasons for decisions to discontinue telehealth or not to adopt the technology. The survey is included as Supporting Information 3.

### 2.2 Analyses

We compared respondents and nonrespondents on observables to identify nonresponse bias and determine if there is a need to weight survey responses. Based on findings (discussed in Sections 3, 4 below), we determined not to weight responses.

We analyzed survey responses to determine percentages of respondents first adopting any telehealth technology by year, or in other words, the percentage of HHAs entering telehealth each year for the first time. We examined the timing of HHAs' adoption and discontinuation of the first technology for each HHA by year: (1) virtual visits (audio only, video visits, and/or therapy sessions via video), (2) remote patient monitoring (RPM; e.g., vital signs, EKG/ECG, and breathing rates), and (3) remote client surveys (including surveys designed to identify specific conditions, screens for diagnosis, and medication adherence) (Supporting Information 2 provides additional detail on how we defined each technology). All are presented as numbers and percentages, where percentages for discontinuation have as denominators the number of respondents who adopted the specific technology, rather than all respondents. Finally, we present reasons for the decision not to adopt or discontinue the use of telehealth technologies.

## 3 Results

We received 791 responses to the survey—a 37% response rate. Except for Census divisions, there were no significant differences at the 0.05 significance level between survey respondents and nonrespondents on any characteristics: size, ownership, percentage of patients with dementia, activities of daily living, and quality—Home Health Consumer Assessment of Healthcare Providers and Systems (HHCAHPS) and Patient Care Star Rating (see Supporting Information 1, Table 1). While the difference in distribution across Census divisions was significant (*p* < 0.001), examination of the percentage differences suggests that they are not meaningfully different.

**FIGURE 1:**
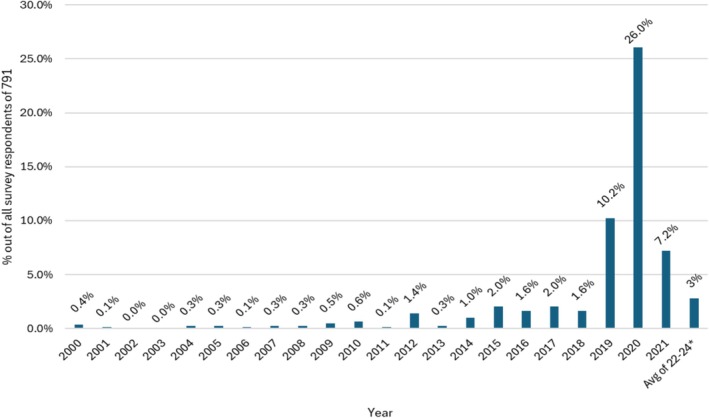
Percentages of home health agencies adopting telehealth for the first time, by year (2000–2024). *Note*: Survey closed in 2024 and some agencies responses may have been for this period.

Figure 1 shows the percentage of HHAs in our sample adopting telehealth for the first time in each year. Prior to 2020, only a minority of HHAs reported first adoption of telehealth in any year, ranging from less than 1% prior to 2011 up to 10.2% by 2019. In 2020, this percentage more than doubled the 2019 rate to 26%. This large adoption rate during 2020 coincided with the first COVID‐19 year. By 2021, the second year of COVID‐19, adoption continued but at a much lower rate of 7.2%. The cumulative percentage of HHAs adopting telehealth by the end of our survey in 2024 reached 64.9%, with the remaining one third of HHAs having never adopted any type of telehealth.

**FIGURE 2:**
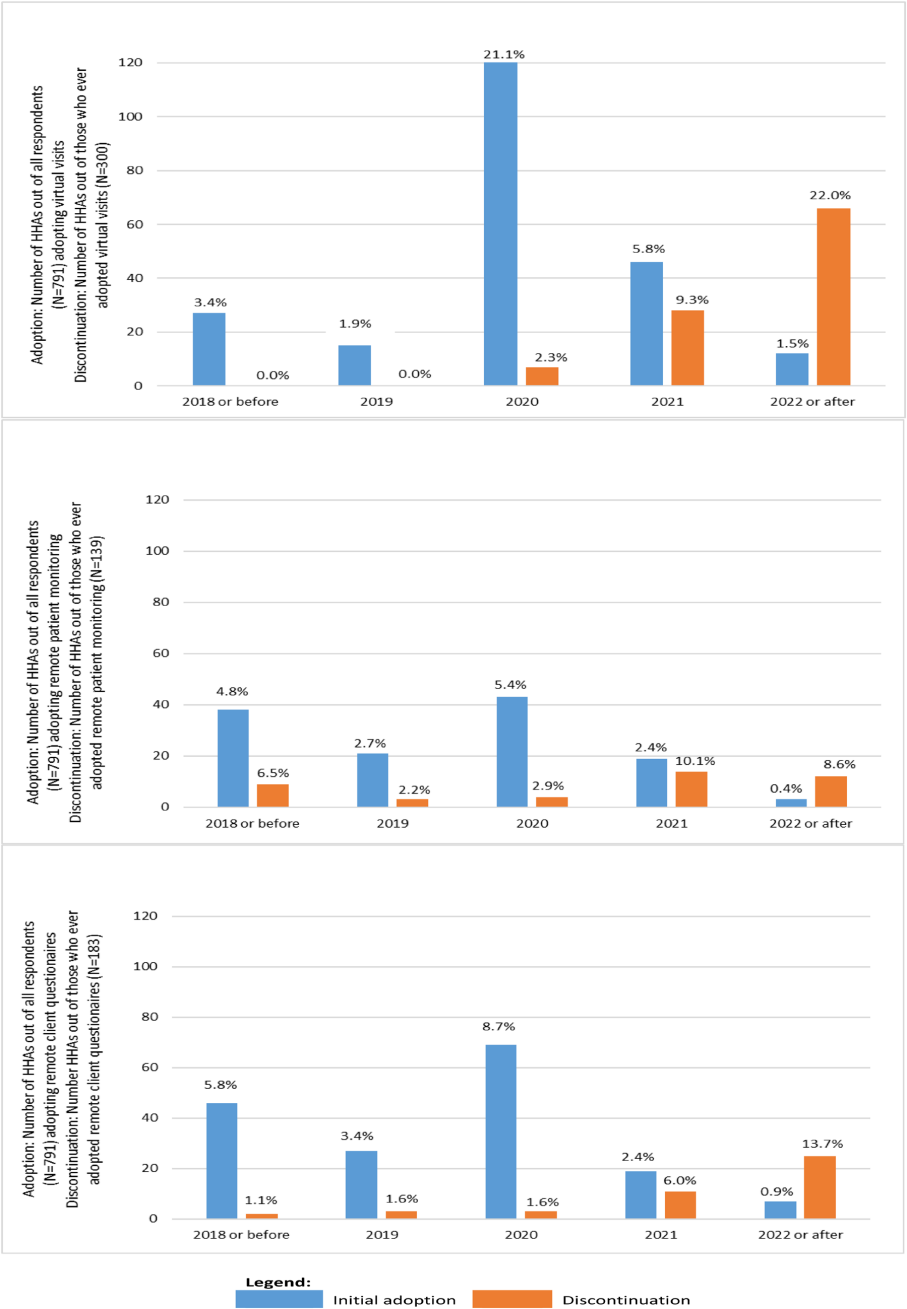
(a) Virtual visits—initial adoption and discontinuation by year. (b) Remote patient monitoring—initial adoption and discontinuation by year. (c) Remote client questionnaires—initial adoption and discontinuation by year. Blue bar = initial adoption. Orange bar = discontinuation.

Figure 2a–c displays the percentage of responding HHAs adopting each type of telehealth technology as their first technology for entry into telehealth by year. They also present the percentage of HHAs discontinuing each technology. Only 3.4% of HHAs had adopted virtual visits between 2000 and 2018, with an additional 1.9% adopting in 2019 (blue bars, Figure 2a). Adoption increased during the COVID‐19 pandemic, with 21.1% and 5.8% of HHAs newly adopting virtual visits in 2020 and 2021, respectively, decreasing to 1.5% of HHAs newly adopting virtual visits from 2022 to 2024. Among HHAs adopting virtual visits, none discontinued by 2019; however, 2.3% of HHAs discontinued virtual visits in 2020, increasing to 9.3% in 2021 and 22.0% in 2022 or later (orange bars, Figure 2a).

Unlike the clear peak in initial adoption of virtual visits, we observed a much smaller increase in the adoption of RPM in 2020, at 5.4%, relative to 2.7% adopting in 2019 (blue bars, Figure 2b). Discontinuation of RPM was limited prior to 2020 but increased to 10.1% of adopting HHAs in 2021 and 8.6% in 2022 and later (orange bars, Figure 2b).

Adoption of remote client surveys was slightly higher than RPM and virtual visits prior to COVID‐19, with 5.8% of HHAs adopting by 2018 and 3.4% adopting in 2019 (blue bars, Figure 2c). However, the increase during COVID‐19 did not reach the magnitude of adoption of virtual visits, peaking at 8.7% in 2020. It then followed a similar pattern as the two other technologies, with fewer adoptions in 2021 and 2022 or later. Similar to the two other technologies, there were few discontinuations prior to 2021, but nearly 20% of adopting HHAs discontinued use between 2021 and 2024 (orange bars, Figure 2c).

**FIGURE 3:**
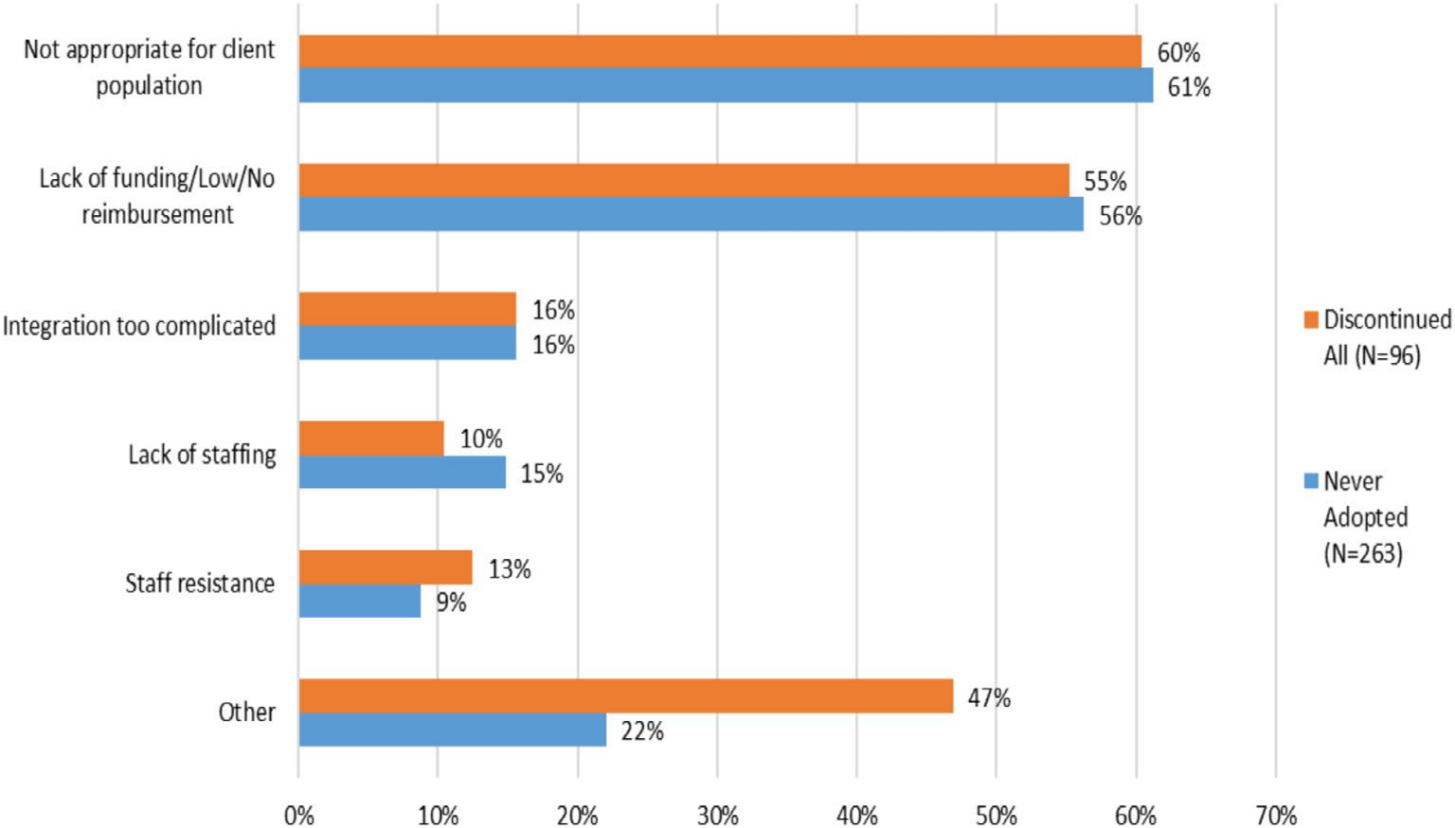
Reasons for never adopting or discontinuing technologies. Orange bar = discontinued all (*N* = 96). Blue bar = never adopted (*N* = 263).

Figure 3 presents the reported reasons for not adopting telehealth among those HHAs that never adopted telehealth (*n* = 263, blue bars) and reasons for discontinuing telehealth among the HHAs that adopted and then discontinued telehealth (*n* = 96, orange bars). Respondents could select more than one reason for not adopting or for discontinuing and could also provide free‐text responses. The most common reason given by both never‐adopting and discontinuing HHAs was that telehealth was not appropriate for their client population, reported by 60% of discontinuing and 61% of never‐adopting HHAs. This reason was also the most common response in the free text with 44% of the never‐adopting and 46% of the discontinuing HHAs. They elaborated that their patients tended to be older adults, often around 80 years old, not technology savvy, and preferred face‐to‐face visits to virtual interactions. In fact, some felt very strongly that home health care cannot and should not be provided virtually as exemplified by one response provided in all capital letters: “WE SEE PATIENTS IN THE HOME”. HHAs also reported that patients shared the belief that home health should not be provided virtually (specifically, this was reported by 18% of never‐adopting HHAs and 10% of discontinuing HHAs). Costs and lack of reimbursement were the second most common concern, also with similar response levels for both discontinuing and never‐adopting HHAs, at 55% and 56% respectively, and were also reported in 10%–20% of free text responses. Another issue raised in the free text responses was that telehealth was adopted only because of COVID‐19 and was discontinued as the pandemic waned and it became clear that Medicare reimbursement was not forthcoming. Other reported issues included difficulties in integrating telehealth into the workflow of the agency, lack of staffing, and resistance by staff, each of which was reported by less than 20% of HHAs.

## 4 Discussion

Our results show that HHAs' use of telehealth was low prior to 2020, but that adoption spiked starting in 2020 with the COVID‐19 pandemic, reaching 65% adoption by HHAs. In particular, adoption of virtual visits increased much more than the two other technologies, presumably as HHAs sought to reduce disease transmission and manage shortages of staff and protective equipment. However, 19% of HHAs adopting telehealth completely discontinued its use by 2024. Furthermore, of the 19% of HHAs discontinuing its use, the majority, 14 percentage points, were those adopting the technology in 2020 or after. This group reportedly did so due to its high cost, lack of Medicare reimbursement, and/or a belief that it may not be appropriate for their patient population under normal, non‐epidemic conditions. Additionally, we found a plurality of HHAs, 33%, that seemed to believe that telehealth has no place in providing home health services at all and chose not to adopt telehealth during our survey period, even in the face of an epidemic with a high level of contagion and strong incentives to avoid home visits.

Thus, our survey found that only about half the HHAs, 53%, can still be counted as users of telehealth at the end of 2024. There is only a small subset of 20% HHAs within this larger group, which views telehealth to be beneficial and complementary to home visits as evidenced by their continued adoption of the technology prior to 2020, the pre‐pandemic period. Possibly, there are additional HHAs within the group that adopted telehealth during the pandemic that see value in it and intend to keep using it.

Indeed, Figure 1 suggests that absent COVID‐19, we may have seen the beginning of a systematic diffusion of telehealth, following the innovation diffusion process proposed by Dearing and Cox [11]. As the authors describe the process, the first to adopt a new technology are the innovators, typically 2.5% of the firms in an industry, followed by the early adopters who are the opinion leaders and usually account for another 13.5% of the firms. The next 34% of firms are the early majority. In Figure 1, we observe a similar percentage of early adopters with close to 13% of HHAs adopting telehealth by 2018. This era is followed by the first large increase in adoption in 2019, of 10% of HHAs. Thus, just before COVID‐19 emerges in early 2020, telehealth reaches a cumulative 23% diffusion—the beginning of the early majority stage of diffusion.

The question is, therefore, whether absent COVID‐19, the diffusion process would have continued. Would the opinion leaders who drive the early majority surge of adoption described by Dearing and Cox have convinced these early adopters and CMS of the potential benefits of telehealth in home health, and would the diffusion have continued to make strides into home health? These are questions that we cannot answer, as COVID‐19 interrupted the process. We do know that a substantial number of HHAs are still using telehealth at the end of 2024, despite the lack of reimbursement by CMS. If reimbursement does not occur, more HHAs may abandon telehealth eventually. If telehealth, however, is cost‐effective and leads to better patient outcomes, as some claim [12], this will be a missed opportunity. Rigorous evaluations of the effects of telehealth in home health on costs and patient outcomes are needed, and if home health telehealth turns out to be cost‐effective, CMS should adopt a reimbursement policy and incentives that support HHAs' use of telehealth technologies, especially as the expenditures of home health are expected to continue to grow by over 10% per year in the coming decade [13].

In addition, future studies should address potential limitations of this study. First, this study took advantage of a survey conducted as part of a larger study focusing on health outcomes for home health patients with Alzheimer's disease and related dementias receiving telehealth services. Hence, our sample targeted HHAs in the top 20th percent of HHAs in terms of patients with dementia. It is, therefore, unclear whether or not the use of telehealth by these HHAs is representative of the industry as a whole. Yet, on average only 33% (standard deviation 8%) of the patients served by these HHAs have dementia (Supporting Information 1, Table 1). The majority of patients do not, and thus organizational decisions regarding telehealth are likely to be dominated by the needs of patients without dementia. Furthermore, in key informant interviews we conducted, it became clear that telehealth is used for dementia patients only when there is a caregiver at home who is the one using the telehealth equipment. Another potential limitation is related to COVID‐19. As we note above, because of the epidemic, we cannot deduce how many of the 53% HHAs that were still using telehealth by the end of 2024 found it to be beneficial and would continue to use it in the long run. Both of these issues should be addressed in future studies to further elucidate the role that telehealth might play in home health and guide future policies vis‐à‐vis telehealth and home health.

## Disclosure

Debra Saliba is an employee of the Veterans Administration. The views presented here do not represent those of the Department of Veterans Affairs.

## Conflicts of Interest

The authors declare no conflicts of interest.

## Supporting information


**Supporting Information 1.** Methods.

**Supporting Information 2.** Telehealth technologies included in survey.

Supporting Information 3.

## Data Availability

Additional studies on this project and data are still ongoing. Data will be made available once they are completed.
