# Supplementary material for: Telehealth Use by Home Health Agencies Before, During, and After COVID‐19
Source: Health Serv Res. 2025 May 22;60(5):e14645. doi: 10.1111/1475-6773.14645 (PMC12461112; doi:10.1111/1475-6773.14645)
Supplement: Supplementary file 1 — Supporting Information 1. Methods. [file HESR-60-0-s003.docx]

**Supplemental Information 1**: Methods

This Research Brief presents an analysis based on data collected by a national survey of 2,135 home health agencies that serve a majority of patients with dementia (on average 33%) conducted during the period 10/2023 –11/2024. The survey was conducted by the Brown University School of Public Health Survey Research Center. The survey included questions about 1) whether or not the agency adopted telehealth services; 2) the types of services adopted (see Appendix 2 for the list of services); 3) years of adoption and discontinuation (in detail by specific technology between 2018 and 2021); 4) reasons for adoptions and discontinuations; 5) information about the respondents. Respondents were offered a $50 incentive, and the mailing included letters of support from both the National Association for Home Care & Hospice and the Forum of State Associations. Respondents were asked to complete the survey online and were provided a short URL and a QR code, along with their login code. They were also provided a link to allow them to view a paper copy of the survey.

Telephone contacts were initiated with non-respondents approximately three weeks after the initial mailing of the recruitment letter to allow sufficient time for the letters to be received by the agencies. The types of subsequent contacts to non-respondents varied based on information available about the participant and/or agency. For example, in addition to mailing address and telephone number, email addresses were available for administrators of some agencies while for others, the participant and/or a gatekeeper was unwilling to provide an email address. The cadence of contacts to nonrespondents was determined by the most recent type of contact. For example, if a request was made to resend the recruitment letter, we waited at least 14 days to recontact the agency. If we spoke with or left a voicemail for the participant or a gatekeeper, we generally waited at least six days between contact attempts unless a call-back was requested. During contact attempts, participants were offered the option of completing the questionnaire by telephone in addition to the web-response option. We attempted up to 10 phone contacts per HHA. This was increased to 13 phone contacts in the summer of 2024.

We had a total of 791 responses for a response rate of 37%.

Table 1 compares respondents to non-respondents on characteristics for which we have information. We do not find significant differences at the 0.05 level between the two groups, except for the distribution across Census divisions. However, inspection of this distribution shows that the differences in most cases are small, except for the South Atlantic.

The data presented in Figures 1-3 in the Research Brief are based on the 791 responses and includes counts and percentages. Each Figure indicates the relevant subsample and its size (i.e. the denominator) in the Y axis label.

**Table 1: Comparison of survey respondents and non-respondents**

|  | **Respondent** |  | **Non-Respondent** |  | **P-value** |
| --- | --- | --- | --- | --- | --- |
|  | N=791 | % | N=1344 | % |  |
| Size |  |  |  |  | 0.579 |
| 0-49 patients | 40 | 5 | 79 | 6 |  |
| 50-99 patients | 128 | 16 | 259 | 19 |  |
| 100-199 patients | 200 | 25 | 340 | 25 |  |
| 200-449 patients | 199 | 25 | 314 | 23 |  |
| 450-749 patients | 85 | 11 | 139 | 10 |  |
| 750-999 patients | 37 | 5 | 64 | 5 |  |
| 1000-2999 patients | 88 | 11 | 132 | 10 |  |
| 3000+ patients | 14 | 2 | 17 | 1 |  |
| Ownership |  |  |  |  | 0.079 |
| For-Profit | 698 | 88 | 1221 | 91 |  |
| Non-Profit | 82 | 10 | 114 | 8 |  |
| Government | 11 | 1 | 9 | 1 |  |
| Census Division |  |  |  |  | <.001 |
| East North Central | 154 | 19 | 209 | 16 |  |
| East South Central | 20 | 3 | 30 | 2 |  |
| Middle Atlantic | 22 | 3 | 38 | 3 |  |
| Mountain | 53 | 7 | 66 | 5 |  |
| New England | 23 | 3 | 17 | 1 |  |
| Pacific | 193 | 24 | 334 | 25 |  |
| South Atlantic | 143 | 18 | 327 | 24 |  |
| West North Central | 25 | 3 | 28 | 2 |  |
| West South Central | 158 | 20 | 295 | 22 |  |
|  |  |  |  |  |  |
|  | **Mean** | **SD** | **Mean** | **SD** |  |
| Percent of patients with ADRD - annual average over 4 years | 33.4 | 8.1 | 34.0 | 8.7 | 0.126 |
|  |  |  |  |  |  |
| Average ADL Score (Range = 0 - 7) | 4.1 | 0.6 | 4.2 | 0.6 | 0.455 |
|  |  |  |  |  |  |
| 2021 CAHPS Survey Summary Star Rating | 3.5 | 1.0 | 3.4 | 1 | 0.126 |
| 2021 Patient Care Star Rating | 3.2 | 1.0 | 3.3 | 1.1 | 0.063 |
