# Supplementary material for: Telehealth Use by Home Health Agencies Before, During, and After COVID‐19
Source: Health Serv Res. 2025 May 22;60(5):e14645. doi: 10.1111/1475-6773.14645 (PMC12461112; doi:10.1111/1475-6773.14645)
Supplement: Supplementary file 2 — Supporting Information 2. Telehealth technologies included in survey. [file HESR-60-0-s001.docx]

**Supplemental Information 2:** Telehealth Technologies Included in Survey

**Virtual Health Care Visits**

1. Virtual health care visits with clients by telephone without video.
2. Virtual health care visits with clients by telephone by videophone or video conference.
3. Virtual therapy-prescribed exercises (e.g. physical, occupational, speech and language) by videophone or videoconference.
4. Other

**Remote Patient Monitoring:**

1. Weight
2. Blood pressure
3. Pulse oximeter
4. Temperature
5. Glucose
6. Monitoring breathing
7. EKG/ECG
8. INR (Prothrombin time test for blood clotting)
9. High quality camera (e.g. enabling wound monitoring)
10. Other

**Remote Client Surveys**

1. Mental health screening
2. Cognitive function testing
3. Surveys about condition-focused symptoms
4. Mobility
5. Medication adherence
6. Other
