# Supplementary material for: Telehealth Use by Home Health Agencies Before, During, and After COVID‐19
Source: Health Serv Res. 2025 May 22;60(5):e14645. doi: 10.1111/1475-6773.14645 (PMC12461112; doi:10.1111/1475-6773.14645)
Supplement: Supplementary file 3 — Supporting Information 3. [file HESR-60-0-s002.docx]

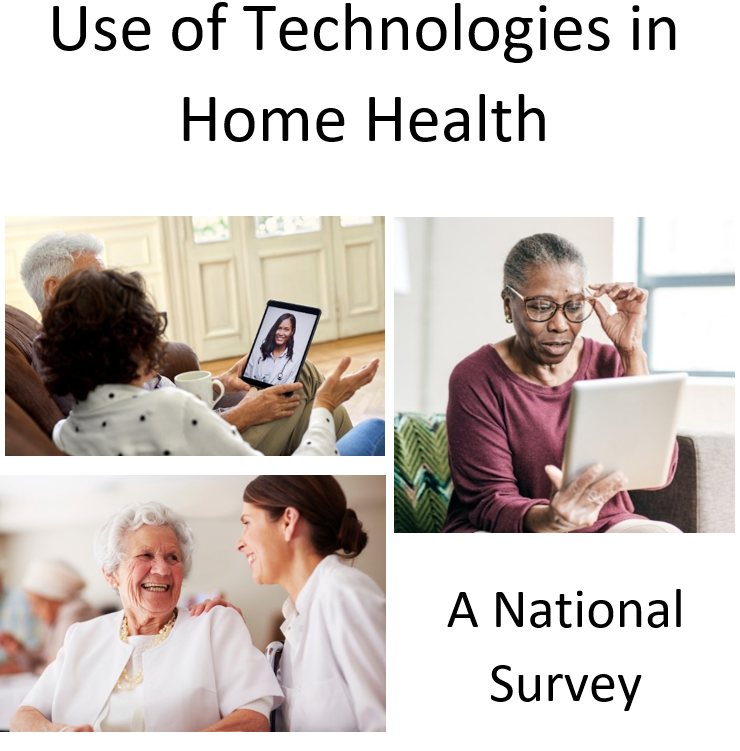


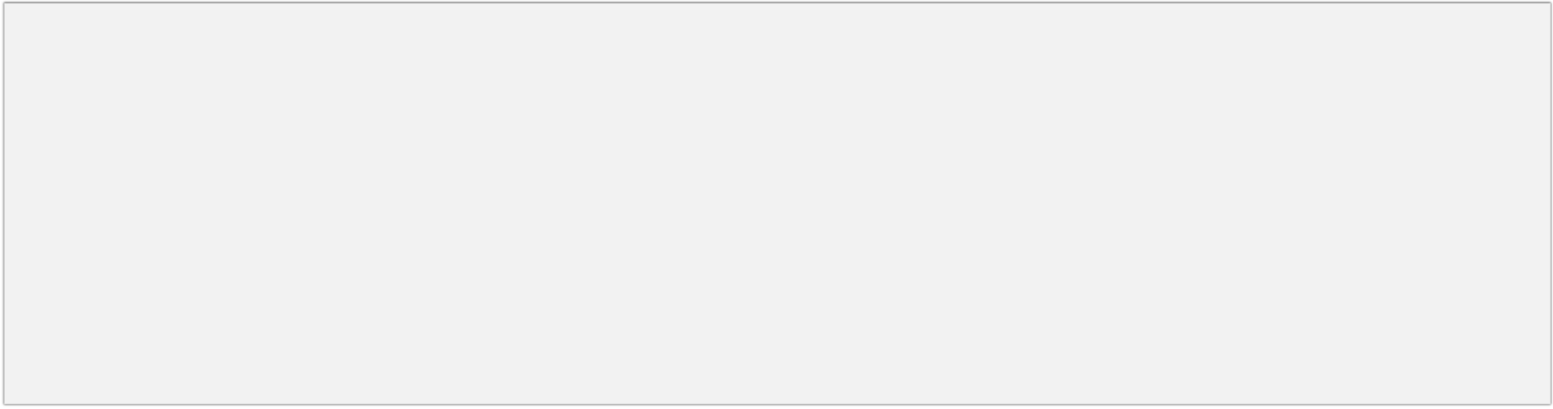


This survey is designed to be answered without having to go to your records. You may consult your colleagues. **Your BEST estimates are OK.** For this survey, we are interested in your clients who are 65 years of age or older (65+).

Please answer each question by marking the box next to your answer. You will sometimes be instructed to skip over some questions. When this happens, the instructions will tell you what question to answer next, like this:

[ ] Yes

[ X ] No *(If No, go to A1)*

**Section A: Opinions about Three Technology Types and Whether Your Agency Uses Them or Not**

The three technology types we include are the following:

- Virtual healthcare visits by phone or videoconference
- Remote Patient Monitoring (RPM): equipment used by clients that can transmit directly to the home care agency for monitoring and potential medical response
- Client questionnaires administered remotely for ongoing monitoring

**A1a** In general, how much do you think the use of these technologies by home health agencies for **clients 65+** has resulted in **TOTAL** (capital and operating) cost savings?

[ ] Not at all

[ ] A little

[ ] Somewhat [ ] Very much

**A1b** In general, how much do you think the use of these technologies by home health agencies for **clients 65+** has improved patient care?

[ ] Not at all

[ ] A little

[ ] Somewhat [ ] Very much

We define **adopting technologies** as: Changing your day‐to‐day operations to use these technologies in the services that you provide to at least some of your home care **clients 65+**.

We define **discontinuing use** as**:** A management decision to stop use of any of these technologies for any reason for any of your home care **clients 65+**.

**A2** Has your agency **EVER** adopted any of the following technologies for **clients 65+**? Please include those that may have been discontinued.

*If any of these are your answers, go to* ***A3*** *on the next page.*

*Please mark all that apply*.

[ ] Virtual healthcare visits by phone or videoconference for clients 65+

[ ] Remote Patient Monitoring (RPM) for clients 65+ (equipment used by clients that can transmit directly to the home care agency for monitoring and potential medical response)

[ ] Client questionnaires administered remotely for ongoing monitoring for clients 65+

[ ] Have NEVER adopted any of the above technologies for clients 65+ (*If NEVER adopted, Go to* ***A2a****)*


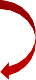


**A2a** Why has your agency **NEVER** adopted any type of the above technologies for **clients 65+**?

*Please mark all that apply*.

[ ] Lack of funding

[ ] Low/No reimbursement for these technologies

[ ] Not appropriate for our client population (e.g., they require complex care, lack of access to internet)

[ ] Lack of staffing

[ ] Integrating use of these technologies into our agency’s workflow/process is too complicated

[ ] Staff resistance

[ ] Other reason (please describe:

***If your agency has NEVER adopted any of these technologies, go to Section F on Page 9. Otherwise, go to A3 on the next page.***

**A3** Approximately during what time period did your agency **FIRST** adopt any of these technologies for **clients 65+**?

[ ] 2018 or before; please indicate what year your agency first adopted any of these technologies for the

**65+ clients** (**YOUR BEST GUESS IS FINE**)

[ ] 2019

[ ] 2020

[ ] 2021

[ ] 2022 or after

**A4** Has your agency discontinued using **ALL** of these technologies for **ALL clients 65+**?

[ ] Yes *(If Yes, go to* ***A4a****)*

[ ] No *(If No, go to* ***Section B*** *on the next page)*

**A4a** Why did your agency discontinue use of **ALL** these technologies for **ALL clients 65+**?

*Please mark all that apply.*

[ ] Low/No reimbursement for these technologies

[ ] Not appropriate for our client population (e.g., they require complex care, lack of access to internet)

[ ] Lack of staffing

[ ] These technologies were too complex to be integrated into our agency workflow/process

[ ] Staff resistance

[ ] Other reason (please describe: )

**A4b** In approximately what year did your agency discontinue use of **ALL** these technologies for **ALL clients 65+**?

[ ] 2018 or before; please indicate what year your agency discontinued all these technologies for the 65+ clients (**YOUR BEST GUESS IS FINE**)

[ ] 2019

[ ] 2020

[ ] 2021

[ ] 2022 or after

## Section B: Virtual Healthcare Visits

**B1** Has your agency **EVER ADOPTED** any type of **virtual health care visits** for clients 65+? [ ] Yes *(If Yes, go to* ***B2****)*

[ ] No *(If No, go to* ***Section C*** *on next page)*

**B2** Please indicate the time frame when the following **virtual healthcare visits** for clients 65+ were **ADOPTED**.

*Mark one answer in each row.*

| Types of Virtual Health Care Visits | Never adopted | **Pre‐COVID** | | **During COVID** | | |
| --- | --- | --- | --- | --- | --- | --- |
|  |  | 2018 or before | 2019 | 2020 | 2021 | 2022 or after |
| 1. Virtual health care visits with clients by telephone without video | [ ] | [ ] | [ ] | [ ] | [ ] | [ ] |
| 1. Virtual health care visits with clients by videophone or video conference | [ ] | [ ] | [ ] | [ ] | [ ] | [ ] |
| 1. Virtual therapy‐prescribed exercises (e.g., physical, occupational, speech and language) by videophone or video conference | [ ] | [ ] | [ ] | [ ] | [ ] | [ ] |
| 1. Other (please describe:   ) | [ ] | [ ] | [ ] | [ ] | [ ] | [ ] |

**B3** Has your agency **DISCONTINUED** any type of **virtual health care visits** for clients 65+? [ ] Yes *(If Yes, go to* ***B4****)*

[ ] No *(If No, go to* ***Section C*** *on next page)*

**B4** Please indicate the time frame when the following **virtual healthcare visits** for clients 65+ were

## DISCONTINUED.

*Mark one answer in each row.*

| Types of Virtual Health Care Visits | Still using or never adopted | **Discontinued the technology** | | | | |
| --- | --- | --- | --- | --- | --- | --- |
|  |  | **Pre‐COVID** | | **During COVID** | | |
|  |  | 2018 or before | 2019 | 2020 | 2021 | 2022 or after |
| 1. Virtual health care visits with clients by telephone without video | [ ] | [ ] | [ ] | [ ] | [ ] | [ ] |
| 1. Virtual health care visits with clients by videophone or video conference | [ ] | [ ] | [ ] | [ ] | [ ] | [ ] |
| 1. Virtual therapy‐prescribed exercises (e.g., physical, occupational, speech and language) by videophone or video conference | [ ] | [ ] | [ ] | [ ] | [ ] | [ ] |
| 1. Other (please describe:   ) | [ ] | [ ] | [ ] | [ ] | [ ] | [ ] |

## Section C: Remote Patient Monitoring (RPM) Technologies

**C1 Remote Patient Monitoring (RPM)** is equipment used by clients that can transmit directly to the home care agency for monitoring and potential medical response**.**

Has your agency **ADOPTED** any type of telehealth **RPM** technologies? [ ] Yes *(If Yes, go to* ***C2****)*

[ ] No *(If No, go to* ***Section D*** *on page 6)*

**C2** Please indicate the time frame when the following telehealth **RPM** technologies for clients 65+ were

## ADOPTED.

*Mark one answer in each row.*

| Types of RPM Technologies | Never adopted | **Pre‐COVID** | | **During COVID** | | |
| --- | --- | --- | --- | --- | --- | --- |
|  |  | 2018 or before | 2019 | 2020 | 2021 | 2022 or after |
| 1. Weight | [ ] | [ ] | [ ] | [ ] | [ ] | [ ] |
| 1. Blood pressure | [ ] | [ ] | [ ] | [ ] | [ ] | [ ] |
| 1. Pulse oximeter | [ ] | [ ] | [ ] | [ ] | [ ] | [ ] |
| 1. Temperature | [ ] | [ ] | [ ] | [ ] | [ ] | [ ] |
| 1. Glucose | [ ] | [ ] | [ ] | [ ] | [ ] | [ ] |

## RPM technologies − continued

*Mark one answer in each row.*

| Types of RPM Technologies | Never adopted | **Pre‐COVID** | | **During COVID** | | |
| --- | --- | --- | --- | --- | --- | --- |
|  |  | 2018 or before | 2019 | 2020 | 2021 | 2022 or after |
| 1. Monitoring breathing (i.e., breath sounds, heart tones) | [ ] | [ ] | [ ] | [ ] | [ ] | [ ] |
| 1. EKG/ECG | [ ] | [ ] | [ ] | [ ] | [ ] | [ ] |
| 1. INR (Prothrombin time test for blood clotting) | [ ] | [ ] | [ ] | [ ] | [ ] | [ ] |
| 1. High‐quality camera (e.g., enabling retinal scan, wound monitoring) | [ ] | [ ] | [ ] | [ ] | [ ] | [ ] |
| 1. Other (please describe:   ) | [ ] | [ ] | [ ] | [ ] | [ ] | [ ] |

**C3** Has your agency **DISCONTINUED** any type of telehealth **RPM** technologies for clients 65+? [ ] Yes *(If Yes, go to* ***C4****)*

[ ] No *(If No, go to* ***Section D*** *on next page)*

**C4** Please indicate the time frame when the following **RPM** technologies for clients 65+ were **DISCONTINUED**.

*Mark one answer in each row.*

| Types of Virtual Health Care Visits | Still using or never adopted | **Discontinued the technology** | | | | |
| --- | --- | --- | --- | --- | --- | --- |
|  |  | **Pre‐COVID** | | **During COVID** | | |
|  |  | 2018 or before | 2019 | 2020 | 2021 | 2022 or after |
| 1. Weight | [ ] | [ ] | [ ] | [ ] | [ ] | [ ] |
| 1. Blood pressure | [ ] | [ ] | [ ] | [ ] | [ ] | [ ] |
| 1. Pulse oximeter | [ ] | [ ] | [ ] | [ ] | [ ] | [ ] |
| 1. Temperature | [ ] | [ ] | [ ] | [ ] | [ ] | [ ] |
| 1. Glucose | [ ] | [ ] | [ ] | [ ] | [ ] | [ ] |

## RPM technologies − continued

*Mark one answer in each row.*

| Types of Virtual Health Care Visits | Still using or never adopted | **Discontinued the technology** | | | | |
| --- | --- | --- | --- | --- | --- | --- |
|  |  | **Pre‐COVID** | | **During COVID** | | |
|  |  | 2018 or before | 2019 | 2020 | 2021 | 2022 or after |
| 1. Monitoring breathing (i.e., breath sounds, heart tones) | [ ] | [ ] | [ ] | [ ] | [ ] | [ ] |
| 1. EKG/ECG | [ ] | [ ] | [ ] | [ ] | [ ] | [ ] |
| 1. INR (Prothrombin time test for blood clotting) | [ ] | [ ] | [ ] | [ ] | [ ] | [ ] |
| 1. High‐quality camera (e.g., enabling retinal scan, wound monitoring) | [ ] | [ ] | [ ] | [ ] | [ ] | [ ] |
| 1. Other (please describe:   ) | [ ] | [ ] | [ ] | [ ] | [ ] | [ ] |

## Section D: Client Questionnaires

**D1** Has your agency **ADOPTED** any **client questionnaires administered remotely for ongoing monitoring** for clients 65+?

[ ] Yes *(If Yes, go to* ***D2****)*

[ ] No *(If No, go to* ***Section E*** *on next page)*

**D2** Please indicate the time frame when the following **client questionnaires administered remotely for ongoing monitoring** for **clients 65+** were **ADOPTED**.

*Mark one answer in each row.*

| Types of Client Questionnaires | Never adopted | **Pre‐COVID** | | **During COVID** | | |
| --- | --- | --- | --- | --- | --- | --- |
|  |  | 2018 or before | 2019 | 2020 | 2021 | 2022 or after |
| 1. Mental health screening | [ ] | [ ] | [ ] | [ ] | [ ] | [ ] |
| 1. Cognitive function testing | [ ] | [ ] | [ ] | [ ] | [ ] | [ ] |
| 1. Surveys about condition‐focused symptoms | [ ] | [ ] | [ ] | [ ] | [ ] | [ ] |
| 1. Mobility | [ ] | [ ] | [ ] | [ ] | [ ] | [ ] |
| 1. Medication adherence | [ ] | [ ] | [ ] | [ ] | [ ] | [ ] |
| 1. Other (please describe:   ) | [ ] | [ ] | [ ] | [ ] | [ ] | [ ] |

**D3** Has your agency **DISCONTINUED** any **client questionnaires administered remotely for ongoing monitoring** for clients 65+?

[ ] Yes *(If Yes, go to* ***D4****)*

[ ] No *(If No, go to* ***Section E*** *on next page)*

**D4** Please indicate the time frame when the following **client questionnaires administered remotely for ongoing monitoring** for clients 65+ were **DISCONTINUED**.

*Mark one answer in each row.*

| Types of Client Questionnaires | Still using or never adopted | **Discontinued the technology** | | | | |
| --- | --- | --- | --- | --- | --- | --- |
|  |  | **Pre‐COVID** | | **During COVID** | | |
|  |  | 2018 or before | 2019 | 2020 | 2021 | 2022 or after |
| 1. Mental health screening | [ ] | [ ] | [ ] | [ ] | [ ] | [ ] |
| 1. Cognitive function testing | [ ] | [ ] | [ ] | [ ] | [ ] | [ ] |
| 1. Surveys about condition‐focused symptoms | [ ] | [ ] | [ ] | [ ] | [ ] | [ ] |
| 1. Mobility | [ ] | [ ] | [ ] | [ ] | [ ] | [ ] |
| 1. Medication adherence | [ ] | [ ] | [ ] | [ ] | [ ] | [ ] |
| 1. Other (please describe:   ) | [ ] | [ ] | [ ] | [ ] | [ ] | [ ] |

## Section E: Clients Receiving Services via Any Technologies

**E1** Please provide estimates of **the overall utilization of all the technologies** for clients 65+ for the different years in your agency. (Please consider all these technologies combined together)

*Please mark one answer in each row.*

| **Year** | **Did not utilize any of these technologies during this year** | **Approximately how many clients 65+ were receiving these technologies during the year?** | | | |
| --- | --- | --- | --- | --- | --- |
|  |  | **Very few**  (Less than 25%) | **Some**  (25−49%) | **Most**  (50−74%) | **Almost all**  (75−100%) |
| 2018 | [ ] | [ ] | [ ] | [ ] | [ ] |
| 2019 | [ ] | [ ] | [ ] | [ ] | [ ] |
| 2020 | [ ] | [ ] | [ ] | [ ] | [ ] |
| 2021 | [ ] | [ ] | [ ] | [ ] | [ ] |
| 2022 | [ ] | [ ] | [ ] | [ ] | [ ] |

**E2** Which of the following types of clients 65+ in your agency currently receive services with and without these technologies?

*Mark at least one answer in each row.*

| **Clients** | **NO** services provided to these types of clients by our agency | Services provided to these types of clients **without any of these technologies** | Services provided to these types of clients **with at least one of these technologies** |
| --- | --- | --- | --- |
| a. With **mild** dementia and **living alone** | [ ] | [ ] | [ ] |
| b. With **mild** dementia and **living with a caregiver** | [ ] | [ ] | [ ] |
| c. With **severe** dementia and **living alone** | [ ] | [ ] | [ ] |
| d. With **severe** dementia and **living with a caregiver** | [ ] | [ ] | [ ] |

## Section F: Questions about You

**F1** What is your position or title in your agency?

**F2** How many years have you worked in this agency? [ ] Less than 1 year

[ ] 1 year but less than 2 years [ ] 2 years but less than 3 years [ ] 3 years but less than 4 years [ ] 4 years but less than 5 years [ ] 5 years but less than 6 years [ ] 6 or more years

# Thank you for your participation!

**Please mail back the questionnaire to:**

**School of Public Health Survey Research Center Brown University**

**Box 1886**

**Providence, RI 02912**
